# Epidemiological Trends and Clinical Characteristics of Epstein–Barr Virus Infection in Febrile Children in Zhejiang, China: A Retrospective Study

**DOI:** 10.1002/iid3.70468

**Published:** 2026-06-03

**Authors:** Hao Zhou, Xuanwen Ru, Wenqin Jin, Simiao Chen, Qing Ye

**Affiliations:** ^1^ Department of Laboratory Medicine The Children's Hospital, Zhejiang University School of Medicine, National Clinical Research Center for Child Health, National Children's Regional Medical Center Hangzhou China; ^2^ School of Medical Technology and Information Engineering Zhejiang Chinese Medical University Hangzhou China

**Keywords:** Epstein–Barr virus, infectious mononucleosis, primary infection, reactivation

## Abstract

**Background:**

This study aimed to investigate the epidemiological trends, age‐ and sex‐related patterns, and clinical and immunological characteristics of Epstein–Barr virus (EBV) infection among febrile children.

**Methods:**

We analyzed EBV DNA data from 9151 febrile children and serological antibody profiles from 6934 cases. A clinical cohort of 377 patients was further evaluated to compare primary infection (*N* = 246) versus reactivation (*N* = 131), as well as infectious mononucleosis (IM, *N* = 134) versus non‐IM (*N* = 112).

**Results:**

EBV DNA positivity peaked in autumn (September–October), whereas VCA‐IgM levels reached their highest in autumn–winter. Primary infection was most prevalent in children aged 1–9 years, peaking in the 3–6‐year age group (58.4%), with higher rates observed in females. Reactivation rates increased with age, reaching 7.8% in children aged ≥ 9 years. Clinically, primary infection is characterized by increased EBV DNA loads, lymphocyte percentages, and atypical lymphocytes and elevated liver enzyme levels. In contrast, reactivation was associated with increased proportions of CD19+ and CD4+ lymphocytes. IM patients had higher lymphocyte percentages, atypical lymphocytes, and liver enzyme abnormalities than non‐IM patients did, who had higher neutrophil percentages and hsCRP levels.

**Conclusion:**

EBV infection in febrile children exhibits distinct seasonal, age‐related, and sex‐related patterns. Primary infection and IM are clinically distinguished by pronounced liver involvement and lymphocytosis, whereas reactivation and non‐IM infections present with different immunological profiles and nonspecific inflammation, providing valuable insights for differential diagnosis and clinical management.

## 1 Introduction

Epstein–Barr virus (EBV), a member of the human herpesvirus family, has attracted considerable attention since its discovery in 1964 because of its close association with a wide range of infectious diseases and malignancies. EBV is among the most prevalent viruses worldwide, with more than 90% of adults estimated to become infected during their lifetime. The virus is characterized by alternating phases of latency and reactivation [1]. Primary infection typically occurs in childhood or adolescence and is often asymptomatic or manifests only as mild respiratory symptoms. However, in some adolescents and young adults, it can lead to the classic clinical syndrome of infectious mononucleosis (IM) [2].

In recent years, the epidemiological pattern of EBV has exhibited new characteristics. On the one hand, there are marked regional differences in the age of primary infection: in developing countries, most children acquire EBV between the ages of 3 and 6 years, whereas in many developed countries, the peak of primary infection is delayed until adolescence, thereby increasing the incidence of IM [3]. On the other hand, EBV is associated with disease risk in immunocompromised individuals. In particular, reactivation of EBV in patients undergoing hematopoietic stem cell transplantation can lead to graft‐versus‐host disease or EBV‐associated posttransplant lymphoproliferative disorder (PTLD) [4]. From a clinical perspective, EBV infection manifests not only as an acute illness but is also causally linked to several malignancies, including nasopharyngeal carcinoma, Hodgkin lymphoma, a subset of gastric cancers, and T/NK‐cell lymphomas [5, 6]. In addition, EBV is thought to contribute to the pathogenesis of autoimmune diseases such as multiple sclerosis and systemic lupus erythematosus [7, 8]. EBV is far from being a simple and common pediatric pathogen; rather, it is a virus with complex clinical significance. Although EBVs have been extensively studied worldwide, comprehensive epidemiological data on febrile pediatric populations in Zhejiang, China, remain limited. In particular, large‐scale analyses distinguishing age distribution, sex differences, and seasonal patterns and differentiating between primary infection and viral reactivation are lacking.

To address this gap, the present study systematically analyzed 9151 children who underwent EBV DNA and serological testing, providing the first in‐depth insights into the epidemiological characteristics of EBV infection in this region. Therefore, a thorough understanding of its epidemiology and clinical manifestations is essential not only for improving early diagnosis and prevention but also for laying the groundwork for future vaccine development and targeted therapies in high‐risk pediatric populations.

## 2 Methods

### 2.1 Study Population and Grouping

**Figure 1:**
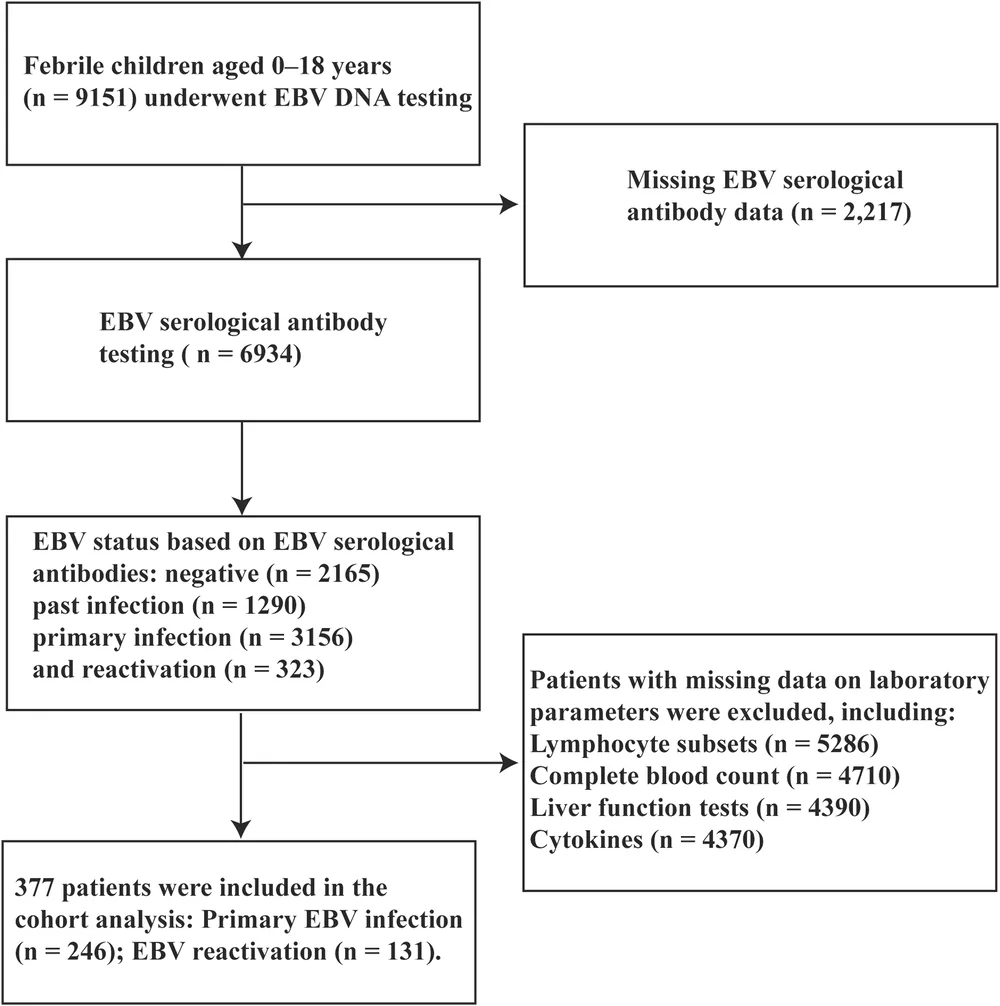
Study flowchart of patient enrollment and grouping.

This study was designed as a retrospective observational study. It included children with fever who were treated at the Children's Hospital, Zhejiang University School of Medicine, and who underwent blood tests from April 2022 to April 2025. We excluded patients with malignancies or immunocompromised states (e.g., congenital immunodeficiencies or long‐term immunosuppressive therapy). A total of 9151 people underwent EBV DNA testing, among whom 2217 did not undergo serum EBV antibody testing. The remaining 6934 patients were classified into 4 groups on the basis of the VCA‐IgM, VCA‐IgG, EA‐IgG, and EBNA‐1‐IgG profiles (uninfected, previous infection, primary infection, and reactivation). After patients whose clinical records were incomplete (e.g., missing CBC data, liver function, lymphocyte subset data, or cytokine data) were excluded, a final cohort of 377 patients was established. The detailed enrollment flowchart is shown in Figure 1.

### 2.2 Diagnosis and Classification of EBV Infection

In this study, serum VCA‐IgM, VCA‐IgG, early antigen (EA)‐IgG, and EBNA‐1‐IgG levels, which represent different pathological stages of EBV infection, were measured [9, 10, 11]. On the basis of combinations of these serological markers, the EBV infection status was classified as follows: *Uninfected:* Negative for EBNA‐1‐IgG, VCA‐IgG, and EA‐IgG. *Primary infection:* Positive for EA‐IgG and/or VCA‐IgM but negative for EBNA‐1‐IgG. *Past infection:* Negative for EA‐IgG and VCA‐IgM but positive for VCA‐IgG and/or EBNA‐1‐IgG. *Reactivation:* Positive for EBNA‐1‐IgG accompanied by positivity for EA‐IgG and/or VCA‐IgM. The diagnostic criteria for IM were as follows: elevated liver enzymes at the first clinical visit (AST ≥ 100 U/L or ALT ≥ 100 U/L) or ≥ 10% atypical lymphocytes and at least three of the following clinical manifestations during the disease course: fever, rash, pharyngitis, cervical lymphadenopathy, hepatomegaly, or splenomegaly [9].

### 2.3 Clinical and Laboratory Data Collection

Clinical information, including sex and age, was collected at the time of the first hospital visit. Laboratory assessments performed during the initial evaluation included white blood cell (WBC) count, lymphocyte (Lym) percentage, neutrophil (Neu) percentage, monocyte (Mon) percentage, eosinophil (Eos) percentage, basophil (Baso) percentage, and atypical lymphocyte percentage. Inflammatory markers included high‐sensitivity C‐reactive protein (hsCRP). Liver function and enzymatic parameters included aspartate aminotransferase (AST), alanine aminotransferase (ALT), lactate dehydrogenase (LDH), alkaline phosphatase (ALP), and total bilirubin (T‐Bil). Immunological measurements revealed cytokines (IL‐2, IL‐4, IL‐6, IL‐10, TNF‐α, and IFN‐γ) and lymphocyte subsets (CD19+, CD3+, CD4+, CD8+, and CD3−CD16+CD56+). Clinical and laboratory data were retrospectively extracted from electronic medical records.

### 2.4 Serological Detection of EBV Antibodies and DNA

Venous blood samples were collected into heparinized tubes and centrifuged to obtain serum. The levels of EBV‐specific antibodies, including VCA‐IgM, VCA‐IgG, EA‐IgG, and EBNA‐1‐IgG, were measured using a chemiluminescence immunoassay (iFlash3000; YHLO; Shenzhen, China). In brief, antibodies in serum bind to EBV antigen‐coated magnetic particles to form complexes. After the addition of acridinium‐labeled anti‐human IgM/IgG, a sandwich complex was formed, and the relative light units were measured using an optical system and converted to antibody concentrations. The interpretation criteria were as follows: VCA‐IgM, VCA‐IgG, EA‐IgG, and EBNA‐1‐IgG concentrations < 20 U/mL, negative; and ≥ 40 U/mL, positive. Equivocal results (20–40 U/mL) were excluded. For EBV DNA detection, DNA was extracted from EDTA‐anticoagulated venous blood using a commercial EBV nucleic acid detection kit (Sansure, Changsha, China). Real‐time quantitative PCR was performed to determine the EBV DNA copy number in serum. The primer and probe sequences have been described previously [12, 13]. The assay detection limit was 400 copies/mL, and samples with ≥ 400 copies/mL EBV DNA were considered positive.

### 2.5 Statistical Analysis

Statistical analyses were performed using R software (version 4.4.1). Categorical variables, such as baseline characteristics and clinical symptoms, are presented as percentages and were compared using the *χ* ^2^ test. Most continuous variables, including laboratory parameters, did not follow a normal distribution and were therefore expressed as medians, with comparisons performed using the Mann–Whitney *U* test. All *p* values were two‐sided, and a *p* < 0.05 was considered to indicate statistical significance.

## 3 Results

### 3.1 Trends in the Positivity Rates of EBV Infection Markers

**Figure 2:**
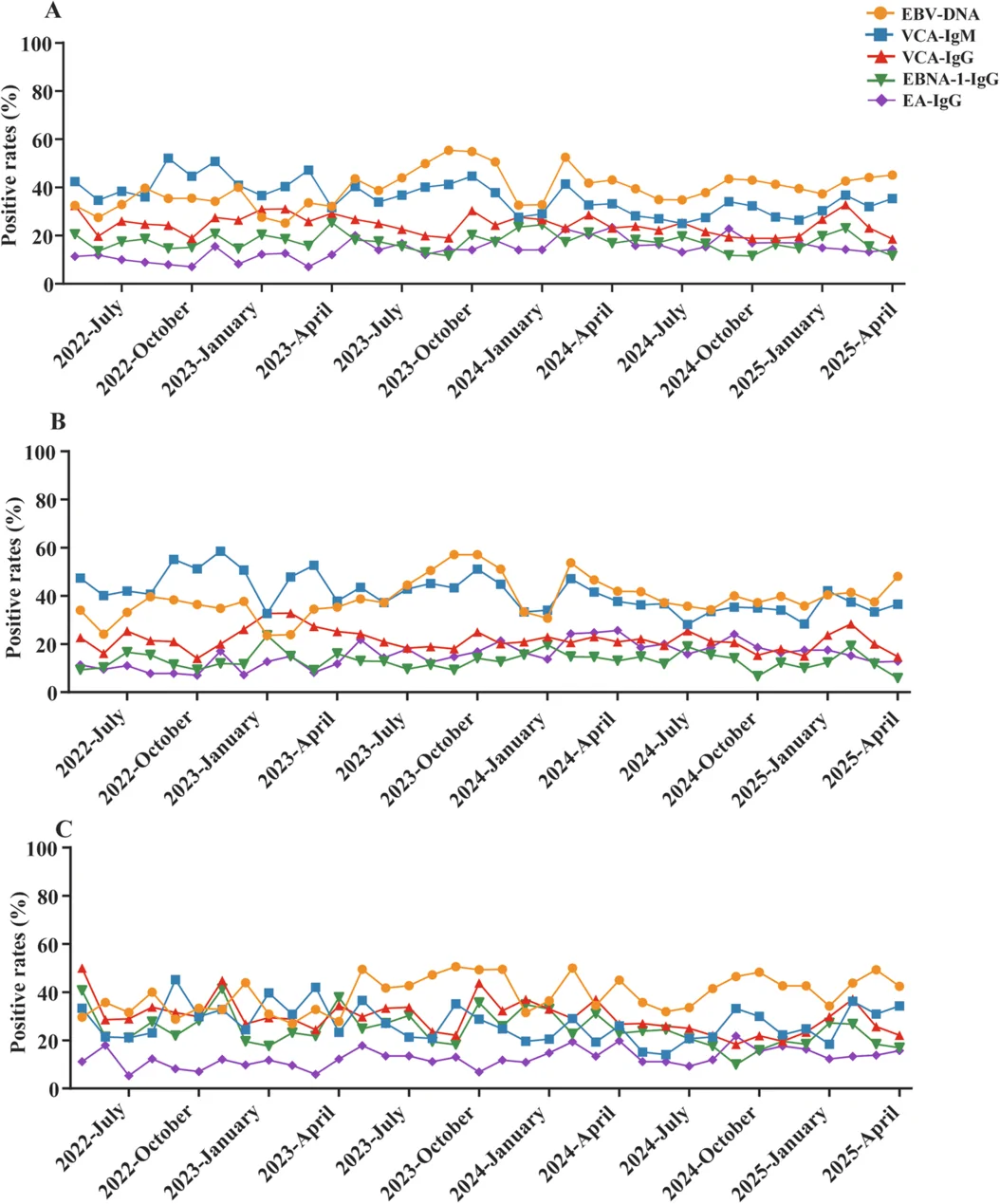
Detection of EBV based on monthly test results for EBV‐specific antibodies and viral DNA from April 2022 to April 2025: (A) overall population, (B) females, and (C) males.

This study included 9151 children who underwent plasma EBV DNA testing, of whom 6934 also received EBV serological antibody testing. The median age of all the enrolled children was 5.3 years, and 51.2% were male. The monthly positivity rates (%) of EBV DNA, VCA‐IgM, VCA‐IgG, EA‐IgG, and EBNA‐1‐IgG are reported in Figure 2 and Table S1. The median positivity rates (with interquartile ranges) were as follows: EBV DNA, 39.5% (34.1%–43.5%); VCA‐IgM, 35.7% (31.3%–40.5%); VCA‐IgG, 24.5% (21.2%–27.0%); EA‐IgG, 14.2% (12.1%–16.8%); and EBNA‐1‐IgG, 17.2% (15.0%–19.8%). Seasonal analysis revealed higher EBV DNA positivity in autumn (September–October; median 43.0%, 43.5%). VCA‐IgM positivity peaked in autumn and winter (September–November; median 41.2%, 44.6%, and 37.8%, respectively), whereas VCA‐IgG and EBNA‐1‐IgG positivity were higher in Winter and Spring (January–February; median 26.8% and 31.1% and 18.5% and 20.3%, respectively).

### 3.2 Age‐Specific Positivity Rates of EBV‐Specific Antibodies and Viral DNA

**Figure 3:**
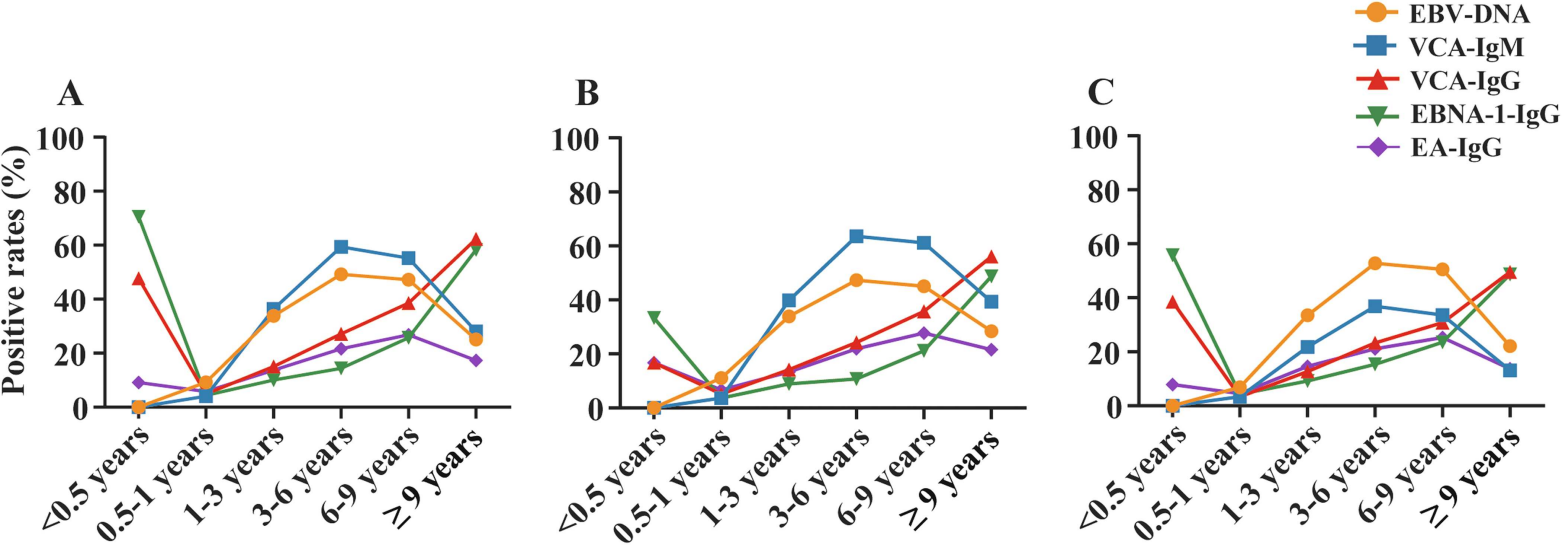
Positive rates of EBV‐specific antibodies and viral DNA across different age and sex groups: (A) overall population, (B) females, and (C) males.

As shown in Figure 3 and Table S2, VCA‐IgM and EBV DNA were undetectable in infants aged < 0.5 years, whereas VCA‐IgG and EBNA‐1‐IgG were markedly higher (47.7% and 70.5%, respectively), with males (52.6% and 76.3%) showing higher rates than females (16.7% and 33.3%). The peak of primary infection occurred between the ages of 1 and 9 years, with VCA‐IgM (59.4%), EA‐IgG (26.8%), and EBV‐DNA (49.2%) reaching their highest levels. In children aged ≥ 9 years, the positivity rates of VCA‐IgG (62.3%) and EBNA‐1‐IgG (58.3%) were the highest and were slightly higher in males (67.9% and 66.8%, respectively) than in females (56.0% and 48.7%, respectively), reflecting the cumulative effect of past infections. Gender differences are notable: females exhibit a higher primary infection rate at ages 3–9 years (EA‐IgG concentration of 26.8% and VCA‐IgM concentration of 63.5%), whereas males show a more significant accumulation of past infections in the early and later stages.

### 3.3 Age Distribution of Primary EBV Infection and Reactivation

**Figure 4:**
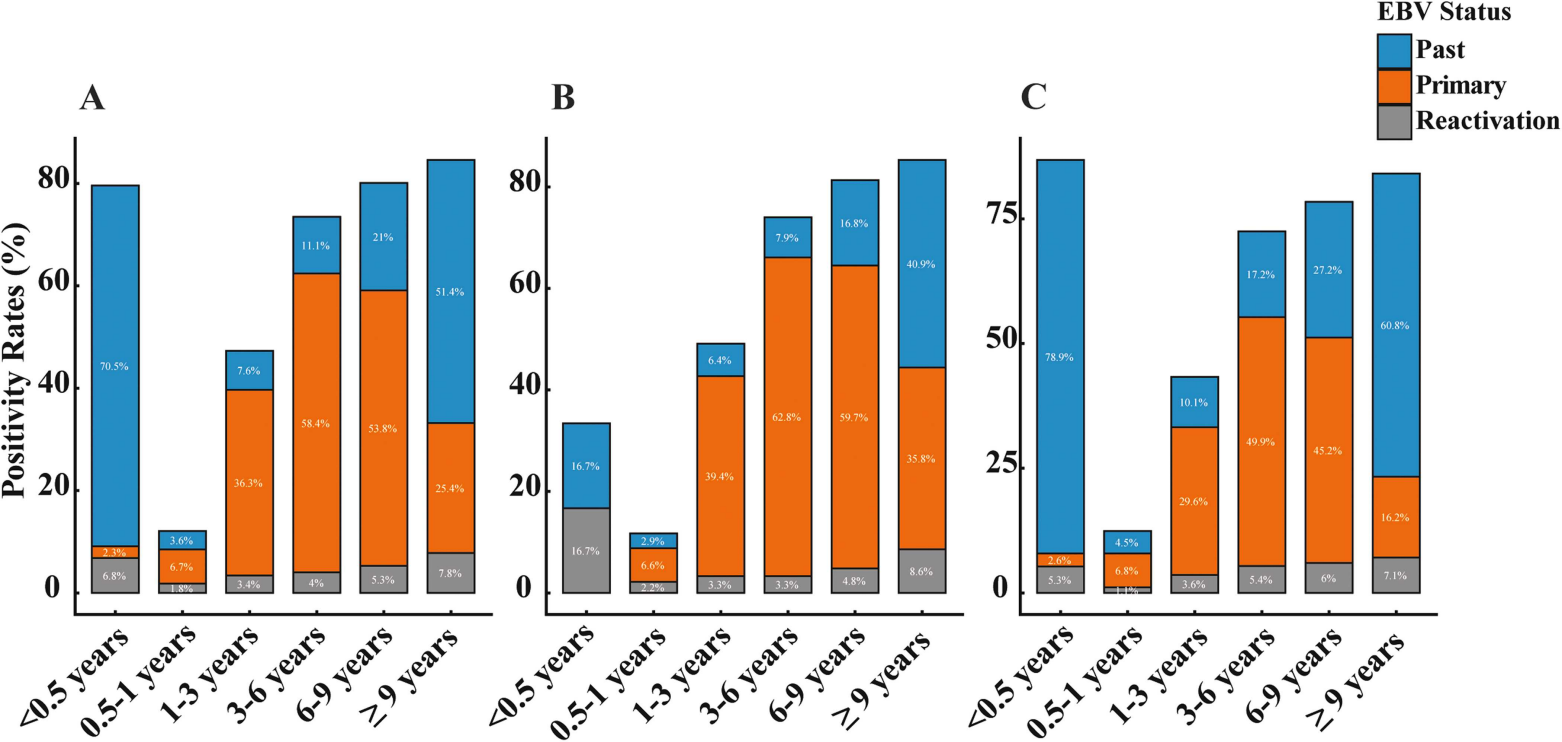
Positive rates of primary EBV infection, past infection, and reactivation across age and sex groups: (A) overall population, (B) females, and (C) males.

Primary infection constituted the predominant category (*n* = 3156), followed by past infection (*n* = 1290) and reactivated infection (*n* = 323). As shown in Figure 4A and Table S3, the prevalence of primary infection increased markedly after 1 year of age, peaking in the 3 to < 6 years age group (58.4%), and subsequently decreased among children aged ≥ 9 years (25.4%). Notably, from the age of 1 year onward, the positivity rate of primary infection remained consistently higher in females than in males (Figure 4B,C). In contrast, the distribution of past infections exhibited a highly characteristic bimodal pattern. High positivity among infants aged < 0.5 years (70.5%) is likely attributable to passive protection conferred by maternally transferred IgG antibodies. As maternal antibodies waned, the rate decreased to its lowest level in the 0.5 to < 1 year age group (3.6%). Thereafter, with increasing age and cumulative natural exposure, the prevalence increased steadily and reached a second peak in the ≥ 9 years group (51.4%). Moreover, after 1 year of age, the prevalence of past infection remained consistently higher in males than in females. In contrast, the overall positivity rate of reactivated EBV infection remained relatively low across all age groups. With the exception of the < 0.5‐year‐old group (6.8%), which may have been influenced by the extremely small sample size and potential interference from maternal antibodies, the reactivation rate also demonstrated a gradual age‐related increase after infancy, reaching the highest level in the ≥ 9‐year‐old group (7.8%). In this group, the rate was slightly higher in females (8.6%) than in males (7.1%), suggesting that as children aged and the population with prior infection expanded, the risk of viral reactivation correspondingly increased.

### 3.4 Comparison of Clinical Characteristics

Significant differences in clinical characteristics were observed between patients with primary and reactivated EBV infections (Table 1). With respect to demographics, while there was no significant difference in median age between the two groups (*p* = 0.063), the sex distribution differed, with a greater proportion of males in the reactivated group (68% vs. 49%, *p* < 0.001). Compared with the reactivated group, the primary group exhibited more robust viral replication and inflammatory responses, characterized by significantly higher EBV DNA loads (5480 vs. 0 copies/mL), WBC counts (14 × 10^9^/L vs. 8 × 10^9^/L), lymphocyte percentages (66% vs. 53%), and atypical lymphocyte percentages (6.0% vs. 0.0%) (all *p* < 0.001). Furthermore, the primary group showed a more pronounced elevation in hepatic biochemical markers, as evidenced by significantly higher levels of ALT, AST, ALP, and LDH (all *p* < 0.001). In terms of the immune response, the primary group had higher levels of the cytokines IL‐10 and IFN‐γ and higher proportions of T cells (CD3+ and CD8+). Conversely, the reactivated group presented significantly greater proportions of CD19 + B cells (12% vs. 4%), CD4+ T cells (31% vs. 15%), and NK cells (CD3−CD16+CD56+) (8.3% vs. 6.6%) (all *p* < 0.001). In contrast, no statistically significant differences in total bilirubin (TBIL) or the levels of IL‐2, IL‐4, IL‐6, or TNF were detected between the two groups.

**Table 1:** Comparison of clinical and laboratory features between primary EBV infection and reactivation.

Characteristic	Overall *N* = 377^a^	Primary *N* = 246^a^	Reactivated *N* = 131^a^	*p* b
Ages (years)	6.0 (4.3, 8.00)	6.0 (4.3, 7.67)	6.3 (4.0, 9.25)	0.063
Genders				< 0.001
Male	209 (55%)	120 (49%)	89 (68%)	
Female	168 (45%)	126 (51%)	42 (32%)	
EBV DNA load (copies/mL)	2410 (0, 13,600)	5480 (1850, 20,900)	277 (122, 397)	< 0.001
WBC (×10^9^/L)	12 (8, 17)	14 (11, 18)	8 (6, 14)	< 0.001
Lym (%)	63 (54, 70)	66 (59, 72)	53 (38, 62)	< 0.001
Neu (%)	26 (18, 37)	23 (16, 30)	38 (28, 51)	< 0.001
Mon (%)	4.2 (3.7, 6.0)	4.0 (3.7, 6.0)	5.2 (3.8, 7.0)	0.003
Eos (%)	0.1 (0.0, 0.8)	0.0 (0.0, 0.4)	0.4 (0.0, 1.4)	< 0.001
Baso (%)	0.0 (0.0, 0.1)	0.0 (0.0, 0.1)	0.1 (0.0, 0.2)	< 0.001
Atypical lymphocytes (%)	5.0 (1.0, 8.0)	6.0 (4.0, 8.0)	0.0 (0.0, 4.0)	< 0.001
hsCRP (mg/L)	6 (2, 13)	6 (3, 13)	4 (0, 14)	0.003
TBIL (μmol/L)	6.4 (5.0, 9.0)	6.5 (5.0, 8.9)	6.2 (5.0, 9.3)	0.8
ALT (U/L)	57 (22, 155)	76 (32, 178)	27 (14, 86)	< 0.001
AST (U/L)	67 (41, 135)	84 (48, 150)	52 (31, 111)	< 0.001
APL (U/L)	220 (168, 277)	229 (174, 293)	192 (156, 252)	< 0.001
LDH (U/L)	517 (429, 701)	552 (457, 715)	449 (293, 664)	< 0.001
IL‐2 (pg/mL)	2.4 (1.8, 3.4)	2.40 (1.8, 3.2)	2.60 (1.9, 3.8)	0.083
IL‐4 (pg/mL)	2.6 (2.0, 3.3)	2.50 (2.0, 3.1)	2.70 (2.1, 3.3)	0.2
IL‐6 (pg/mL)	18 (10, 36)	18 (11, 37)	17 (8, 32)	0.10
IL‐10 (pg/mL)	15 (6, 31)	19 (9, 37)	6 (4, 17)	< 0.001
TNF (pg/mL)	2.1 (1.4, 3.3)	2.10 (1.4, 3.2)	2.20 (1.5, 3.4)	0.7
IFN‐γ (pg/mL)	8 (5, 13)	9 (5, 14)	5 (3, 11)	< 0.001
CD19 (%)	5 (3, 11)	4 (2, 6)	12 (5, 18)	< 0.001
CD3 (%)	83 (77, 87)	85 (80, 89)	78 (71, 83)	< 0.001
CD4 (%)	18 (12, 28)	15 (11, 20)	31 (19, 37)	< 0.001
CD8 (%)	53 (39, 64)	58 (49, 66)	32 (27, 53)	< 0.001
CD3−CD16+CD56+ (%)	7.0 (4.9, 9.8)	6.6 (4.8, 8.5)	8.3 (5.2, 11.9)	0.003

^a^ Median (Q1, Q3); *n* (%).

^b^ Wilcoxon rank sum test; Pearson's *χ* ^2^ test.

Among 246 patients with primary EBV infection, we compared the characteristics of those with IM (*n* = 140) and those without IM (non‐IM, *n* = 119) (Table 2). Although no significant differences in age, sex distribution, or EBV DNA load were detected, the two groups were distinguished by their immune cell profiles or biochemical markers. Specifically, the IM group had higher percentages of lymphocytes (69% vs. 63%) and atypical lymphocytes, whereas the non‐IM group had significantly higher neutrophil percentages (28% vs. 20%) and hsCRP levels (8 vs. 5 mg/L) (all *p* < 0.01). In addition, compared with the non‐IM group, the IM group demonstrated more pronounced increases in hepatic biochemical markers, with ALT, AST, ALP, and LDH levels being higher (all *p* < 0.001). In terms of lymphocyte subsets, the IM group was characterized by a greater percentage of CD8+ T cells (60% vs. 55%), whereas the non‐IM group presented relatively higher proportions of CD19+ B cells (4.23% vs. 3.15%) and CD4+ T cells (16% vs. 14%) (all *p* < 0.05). No significant differences were observed between the two groups in terms of major cytokine levels.

**Table 2:** Comparison of clinical and laboratory features between IM and non‐IM.

Characteristic	Overall *N* = 246^a^	IM *N* = 134^a^	non‐IM *N* = 112^a^	*p* b
Ages (years)	6.0 (4.3, 7.7)	6.0 (4.4, 8.0)	6.0 (4.3, 7.1)	0.3
Genders				0.2
Male	120 (49%)	60 (45%)	60 (54%)	
Female	126 (51%)	74 (55%)	52 (46%)	
EBV DNA load (copies/mL)	5480 (1850, 20,900)	5575 (1880, 20,900)	5480 (1840, 19,550)	0.8
WBC (x10^9^/L)	13.8 (11.2, 17.7)	14.3 (10.7, 18.5)	13.5 (11.5, 17.0)	0.4
Lym (%)	66 (59, 72)	69 (62, 74)	63 (56, 70)	< 0.001
Neu (%)	23 (16, 30)	20 (15, 26)	28 (20, 36)	< 0.001
Mon (%)	4.0 (3.7, 6.0)	4.0 (4.0, 6.0)	4.0 (3.5, 6.0)	0.3
Eos (%)	0.0 (0.0, 0.4)	0.0 (0.0, 0.2)	0.0 (0.0, 0.6)	0.007
Baso (%)	0.0 (0.0, 0.1)	0.0 (0.0, 0.0)	0.0 (0.0, 0.1)	0.087
Atypical lymphocytes (%)	6.0 (4.0, 8.0)	6.0 (5.0, 10.0)	6.0 (3.0, 7.0)	< 0.001
hsCRP (mg/L)	6 (3, 13)	5 (3, 10)	8 (4, 18)	0.002
TBIL (μmol/L)	6.5 (5.0, 8.9)	6.7 (5.0, 9.8)	6.3 (5.0, 7.8)	0.064
ALT (U/L)	76 (32, 178)	162 (106, 314)	33 (22, 57)	< 0.001
AST (U/L)	84 (48, 150)	139 (99, 231)	48 (37, 61)	< 0.001
APL (U/L)	229 (174, 293)	270 (213, 393)	197 (157, 230)	< 0.001
LDH (U/L)	552 (457, 715)	616 (505, 781)	486 (430, 604)	< 0.001
IL‐2 (pg/mL)	2.4 (1.8, 3.2)	2.4 (1.9, 3.4)	2.4 (1.6, 3.0)	0.3
IL‐4 (pg/mL)	2.5 (2.0, 3.1)	2.6 (2.1, 3.3)	2.4 (1.9, 3.0)	0.12
IL‐6 (pg/mL)	18 (11, 37)	16 (10, 36)	21 (13, 38)	0.10
IL‐10 (pg/mL)	19 (9, 37)	21 (10, 41)	19 (8, 35)	0.11
TNF (pg/mL)	2.1 (1.4, 3.2)	2.1 (1.4, 3.4)	2.1 (1.4, 3.2)	0.7
IFN‐γ (pg/mL)	9 (5, 14)	9 (6, 14)	8 (5, 13)	0.2
CD19 (%)	3.7 (2.4, 5.5)	3.2 (2.1, 4.8)	4.2 (3.1, 7.0)	< 0.001
CD3 (%)	85 (80, 89)	85 (81, 89)	84 (80, 88)	0.4
CD4 (%)	15 (11, 20)	14 (11, 20)	16 (12, 21)	0.018
CD8 (%)	58 (49, 66)	60 (50, 68)	55 (47, 65)	0.027
CD3−CD16+CD56+ (%)	6.6 (4.8, 8.5)	6.0 (4.6, 8.1)	7.1 (5.0, 9.0)	0.062

^a^ Median (Q1, Q3); *n* (%).

^b^ Wilcoxon rank sum test; Pearson's *χ* ^2^ test.

## 4 Discussion

In this study, on the basis of 9151 pediatric cases tested for EBV DNA and serology, the age distribution, sex differences, and seasonal characteristics of EBV infection were systematically analyzed, and the epidemiological and clinical features of primary infection and reactivation were explored. The results demonstrate distinct staging and population differences in EBV infection among children in Zhejiang Province, China.

The age distribution in this study underscores a clear transition from passive immunity to active infection and eventual latency. We found that children aged 1–9 years represent the peak period for primary EBV infection, while those aged ≥ 9 years predominantly exhibit past infections. This pattern aligns with previous research indicating that primary exposure is most common in preschool and school‐aged children, whereas older children and adolescents transition into latent states [14, 15]. Notably, only 2.3% of the infants aged < 0.5 years had primary infections despite high VCA‐IgG (47.7%) and EBNA‐1‐IgG (70.5%) positivity. This confirms that the low infection rate in early infancy is not due to a lack of exposure but is instead conferred by the transient protective role of maternally transferred antibodies [16, 17]. The rapid decline in the expression of these markers to their lowest point in the 0.5 to < 1‐year‐old group marked the window in which children became susceptible to their first natural encounter with the virus.

Our analysis revealed significant sex differences in immune responses. From age 1 onward, females exhibited a consistently higher rate of primary infection (peaking at 62.8%), whereas males showed higher cumulative past infections and reactivation rates. While adult studies often suggest that females mount more robust long‐term IgG responses [18], our pediatric data indicate that the timing and nature of these responses diverge early. The higher VCA‐IgM levels in females than in males may be influenced by a complex interplay of socioeconomic behavioral factors (e.g., varying close‐contact patterns) [19] or hormonal effects on the immune system [20]. These findings suggest that male children may require more vigilant long‐term monitoring for EBV‐related complications as they age.

The seasonal distribution of EBV markers reflects the natural course of infection and environmental susceptibility. We observed that EBV DNA and VCA‐IgM positivity peaked during autumn and winter, which coincided with the high‐incidence season for respiratory viruses. During these months, the integrity of children's respiratory immune barriers is often compromised, potentially lowering the threshold for primary EBV infection or the reactivation of latent virus [21, 22]. Conversely, the prevalence of VCA‐IgG and EBNA‐1‐IgG peaked in the winter and spring. This “seasonal lag” is consistent with the typical seroconversion timeline, where specific IgG antibodies reach detectable peaks weeks to months after the acute phase. Furthermore, the return to school and childcare facilities in autumn facilitates increased close contact, which likely drives the initial surge in acute markers followed by the convalescent antibody peaks observed in the subsequent months [23, 24].

Our findings demonstrate that primary EBV infection elicits a significantly more robust inflammatory response than reactivation does, characterized by markedly higher viral loads, atypical lymphocytosis, and elevated hepatic markers. The high EBV DNA loads in the primary group reflect active viral replication within B cells, which serves as a marker of replication activity and as an important determinant of disease severity and hospitalization risk in pediatric populations [25, 26]. Some studies have shown that primary EBV infection can trigger a pronounced T‐cell–mediated immune response, which contributes to atypical lymphocytosis and liver enzyme elevation, particularly in children and adolescents [15, 27]. Furthermore, the significant elevation in ALT and AST levels confirms that the liver remains a primary target organ during acute EBV infection, which aligns with previous reports of EBV‐associated hepatitis in which immune‐mediated damage plays a central role [28, 29].

Among patients with primary EBV infection, those with IM had higher lymphocyte percentages, atypical lymphocyte counts, and liver enzyme levels. These findings reflect the classic features of IM, characterized by a strong T‐cell–mediated immune response and prominent hepatic involvement. Previous studies have shown that IM is frequently associated with lymphocytosis, atypical lymphocyte proportions exceeding 10%, and markedly elevated liver enzymes, particularly in adolescents and young adults [30, 31]. In the present study, the elevated ALT and AST levels observed in the IM group are consistent with reports of EBV‐associated acute hepatitis [32], further supporting the liver as a major target organ in the IM. In contrast, the non‐IM group displayed higher neutrophil percentages, hsCRP levels, and CD19^+^ B‐cell proportions, which are potentially associated with enhanced nonspecific inflammation and a higher proportion of circulating CD19^+^ B cells. This pattern may reflect the heterogeneous clinical presentations of non‐IM EBV infection, ranging from mild to asymptomatic cases. Previous reports suggest that non‐IM infections may present with nonspecific febrile illness or mild inflammatory responses and that B‐cell (CD19^+^) activation is closely linked to latent viral replication within B cells [33]. The elevated hsCRP levels observed in the non‐IM group further support the presence of an inflammatory state, which may correspond to subclinical manifestations of EBV infection.

Despite the clear differences in cellular profiles, we observed no significant differences in most cytokines (IL‐2, IL‐4, IL‐10, IL‐6, TNF‐α). Previous studies have reported that the cytokine response in the IM is typically characterized by elevated IL‐6 and IL‐10 levels [30]; however, such differences were not observed in the present study. This lack of divergence may be attributed to several factors. First, the timing of sampling is critical; cytokine responses are extremely dynamic [34] and may have peaked and subsided before clinical presentation and testing. Second, clinical heterogeneity within the cohorts may mask specific cytokine signatures. Finally, the sensitivity of standard assays may not capture subtle local cytokine fluctuations in the lymphoid tissues where EBV primarily replicates. Future studies using longitudinal sampling and high‐sensitivity multiplex assays are warranted to better delineate the cytokine dynamics of pediatric EBV infection.

Several limitations of this study should be noted. First, as a single‐center retrospective study conducted at a specific tertiary hospital, our cohort primarily included patients with apparent symptoms, potentially excluding mild or asymptomatic community cases. This selection bias may limit the generalizability of our findings to the broader pediatric population. Second, the cross‐sectional and descriptive nature of our data means that dynamic follow‐up and longitudinal immunological validation were lacking. For example, we did not perform functional immune assays—such as B‐cell proliferation or specific activation markers—to complement our cell subset observations; therefore, these immunological findings remain exploratory and require further validation. Future multicenter prospective studies are warranted to mitigate these biases and encompass more diverse demographic data. Longitudinal monitoring and molecular functional analyses should be performed to further elucidate the natural history of EBV infection and the complex, dynamic interactions between the virus and host immune system across various clinical contexts.

In summary, our study highlights the dynamic patterns of EBV infection in children, indicating a clear shift from maternal antibody protection to active infection as they age. In addition to confirming typical age and seasonal trends, we identified clinical and immunological differences between the primary and reactivated states. Furthermore, the distinct immune responses observed in IM versus non‐IM patients suggest that disease severity may be shaped more by the host's immune reaction than by viral load alone. These insights not only improve our understanding of EBV–host interactions but also support more precise diagnosis and personalized care for pediatric patients.

## Author Contributions

**Hao Zhou:** conceptualization, software, methodology, visualization, writing – original draft, writing – review and editing, formal analysis. **Xuanwen Ru:** conceptualization, methodology, software, writing – original draft, writing – review and editing. **Wenqin Jin:** methodology, data curation, validation. **Simiao Chen:** data curation, validation. **Qing Ye:** conceptualization, writing – review and editing, supervision, project administration. All the authors approved the final manuscript.

## Funding

The authors have nothing to report.

## Ethics Statement

The study was conducted in accordance with the Declaration of Helsinki. It was authorized by the Committee on Ethics at Children's Hospital, Zhejiang University School of Medicine (2025‐IRB‐0158‐P‐01).

## Consent

The requirement for informed consent was waived by the Ethics Committee due to the retrospective nature of the study and the use of de‐identified data.

## Conflicts of Interest

The authors declare no conflicts of interest.

## Supporting information

Supporting File.

## Data Availability

The data that support the findings of this study are available from the corresponding author upon reasonable request.
