# Supplementary material for: Epidemiological Trends and Clinical Characteristics of Epstein–Barr Virus Infection in Febrile Children in Zhejiang, China: A Retrospective Study
Source: Immun Inflamm Dis. 2026 Jun 3;14(6):e70468. doi: 10.1002/iid3.70468 (PMC13581078; doi:10.1002/iid3.70468)
Supplement: Supplementary file 1 [file IID3-14-e70468-s001.docx]

**Table S1:** The monthly distribution and positive rate of serum EBV antibodies and plasma EBV DNA.

| **Date** | **EBV-DNA (%)** | | | **VCA-IgM (%)** | | | **VCA-IgG (%)** | | | **EBNA-1-IgG (%)** | | | **EA-IgG (%)** | | |
| --- | --- | --- | --- | --- | --- | --- | --- | --- | --- | --- | --- | --- | --- | --- | --- |
|  | **Female** | **Male** | **Total** | **Female** | **Male** | **Total** | **Female** | **Male** | **Total** | **Female** | **Male** | **Total** | **Female** | **Male** | **Total** |
| 2022-May | 33/97 (34.0) | 16/54 (29.6) | 49/151 (32.5) | 46/97 (47.4) | 18/54 (33.3) | 64/151 (42.4) | 22/97 (22.7) | 27/54 (50.0) | 49/151 (32.5) | 9/97 (9.3) | 22/54 (40.7) | 31/151 (20.5) | 11/97 (11.3) | 6/54 (11.1) | 17/151 (11.3) |
| 2022-June | 33/137 (24.1) | 20/56 (35.7) | 53/193 (27.5) | 55/137 (40.1) | 12/56 (21.4) | 67/193 (34.7) | 22/137 (16.1) | 16/56 (28.6) | 38/193 (19.7) | 14/137 (10.2) | 12/56 (21.4) | 26/193 (13.5) | 13/137 (9.5) | 10/56 (17.9) | 23/193 (11.9) |
| 2022-Jul | 60/181 (33.1) | 12/38 (31.6) | 72/219 (32.9) | 76/181 (42.0) | 8/38 (21.1) | 84/219 (38.4) | 46/181 (25.4) | 11/38 (28.9) | 57/219 (26.0) | 30/181 (16.6) | 8/38 (21.1) | 38/219 (17.4) | 20/181 (11.0) | 2/38 (5.3) | 22/219 (10.0) |
| 2022-Aug | 72/182 (39.6) | 26/65 (40.0) | 98/247 (39.7) | 74/182 (40.7) | 15/65 (23.1) | 89/247 (36.0) | 39/182 (21.4) | 22/65 (33.8) | 61/247 (24.7) | 28/182 (15.4) | 18/65 (27.7) | 46/247 (18.6) | 14/182 (7.7) | 8/65 (12.3) | 22/247 (8.9) |
| 2022-Sep | 64/167 (38.3) | 21/73 (28.8) | 85/240 (35.4) | 92/167 (55.1) | 33/73 (45.2) | 125/240 (52.1) | 35/167 (21.0) | 23/73 (31.5) | 58/240 (24.2) | 19/167 (11.4) | 16/73 (21.9) | 35/240 (14.6) | 13/167 (7.8) | 6/73 (8.2) | 19/240 (7.9) |
| 2022-Oct | 47/129 (36.4) | 19/57 (33.3) | 66/186 (35.5) | 66/129 (51.2) | 17/57 (29.8) | 83/186 (44.6) | 18/129 (14.0) | 17/57 (29.8) | 35/186 (18.8) | 12/129 (9.3) | 16/57 (28.1) | 28/186 (15.1) | 9/129 (7.0) | 4/57  (7.0) | 13/186 (7.0) |
| 2022-Nov | 47/135 (34.8) | 19/58 (32.8) | 66/193 (34.2) | 79/135 (58.5) | 19/58 (32.8) | 98/193 (50.8) | 27/135 (20.0) | 26/58 (44.8) | 53/193 (27.5) | 16/135 (11.9) | 24/58 (41.4) | 40/193 (20.7) | 23/135 (17.0) | 7/58 (12.1) | 30/193 (15.5) |
| 2022-Dec | 26/69 (37.7) | 18/41 (43.9) | 44/110 (40.0) | 35/69 (50.7) | 10/41 (24.4) | 45/110 (40.9) | 18/69 (26.1) | 11/41 (26.8) | 29/110 (26.4) | 8/69 (11.6) | 8/41 (19.5) | 16/110 (14.5) | 5/69 (7.2) | 4/41 (9.8) | 9/110 (8.2) |
| 2023-Jan | 13/55 (23.6) | 21/68 (30.9) | 34/123 (27.6) | 18/55 (32.7) | 27/68 (39.7) | 45/123 (36.6) | 18/55 (32.7) | 20/68 (29.4) | 38/123 (30.9) | 13/55 (23.6) | 12/68 (17.6) | 25/123 (20.3) | 7/55 (12.7) | 8/68 (11.8) | 15/123 (12.2) |
| 2023-Febr | 16/67 (23.9) | 14/52 (26.9) | 30/119 (25.2) | 32/67 (47.8) | 16/52 (30.8) | 48/119 (40.3) | 22/67 (32.8) | 15/52 (28.8) | 37/119 (31.1) | 10/67 (14.9) | 12/52 (23.1) | 22/119 (18.5) | 10/67 (14.9) | 5/52 (9.6) | 15/119 (12.6) |
| 2023-Mar | 38/110 (34.5) | 39/119 (32.8) | 77/229 (33.6) | 58/110 (52.7) | 50/119 (42.0) | 108/229 (47.2) | 30/110 (27.3) | 29/119 (24.4) | 59/229 (25.8) | 10/110 (9.1) | 26/119 (21.8) | 36/229 (15.7) | 9/110 (8.2) | 7/119 (5.9) | 16/229 (7.0) |
| 2023-Apr | 42/119 (35.3) | 25/90 (27.8) | 67/209 (32.1) | 45/119 (37.8) | 21/90 (23.3) | 66/209 (31.6) | 30/119 (25.2) | 31/90 (34.4) | 61/209 (29.2) | 19/119 (16) | 34/90 (37.8) | 53/209 (25.4) | 14/119 (11.8) | 11/90 (12.2) | 25/209 (12.0) |
| 2023-May | 48/124 (38.7) | 50/101 (49.5) | 98/225 (43.6) | 54/124 (43.5) | 37/101 (36.6) | 91/225 (40.4) | 30/124 (24.2) | 30/101 (29.7) | 60/225 (26.7) | 16/124 (12.9) | 25/101 (24.8) | 41/225 (18.2) | 27/124 (21.8) | 18/101 (17.8) | 45/225 (20.0) |
| 2023-June | 73/196 (37.2) | 40/96 (41.7) | 113/292 (38.7) | 73/196 (37.2) | 26/96 (27.1) | 99/292 (33.9) | 41/196 (20.9) | 32/96 (33.3) | 73/292 (25.0) | 25/196 (12.8) | 26/96 (27.1) | 51/292 (17.5) | 28/196 (14.3) | 13/96 (13.5) | 41/292 (14.0) |
| 2023-July | 102/229 (44.5) | 38/89 (42.7) | 140/318 (44.0) | 98/229 (42.8) | 19/89 (21.3) | 117/318 (36.8) | 42/229 (18.3) | 30/89 (33.7) | 72/318 (22.6) | 22/229 (9.6) | 27/89 (30.3) | 49/318 (15.4) | 41/229 (17.9) | 12/89 (13.5) | 53/318 (16.7) |
| 2023-Aug | 139/275 (50.5) | 34/72 (47.2) | 173/347 (49.9) | 124/275 (45.1) | 15/72 (20.8) | 139/347 (40.1) | 52/275 (18.9) | 17/72 (23.6) | 69/347 (19.9) | 31/275 (11.3) | 14/72 (19.4) | 45/347 (13.0) | 34/275 (12.4) | 8/72 (11.1) | 42/347 (12.1) |
| 2023-Sep | 124/217 (57.1) | 39/77 (50.6) | 163/294 (55.4) | 94/217 (43.3) | 27/77 (35.1) | 121/294 (41.2) | 39/217 (18.0) | 17/77 (22.1) | 56/294 (19.0) | 20/217 (9.2) | 14/77 (18.2) | 34/294 (11.6) | 32/217 (14.7) | 10/77 (13.0) | 42/294 (14.3) |
| 2023-Oct | 105/184 (57.1) | 36/73 (49.3) | 141/257 (54.9) | 94/184 (51.1) | 21/73 (28.8) | 115/257 (44.7) | 46/184 (25.0) | 32/73 (43.8) | 78/257 (30.4) | 26/184 (14.1) | 26/73 (35.6) | 52/257 (20.2) | 31/184 (16.8) | 5/73 (6.8) | 36/257 (14.0) |
| 2023-Nov | 89/174 (51.1) | 46/93 (49.5) | 135/267 (50.6) | 78/174 (44.8) | 23/93 (24.7) | 101/267 (37.8) | 35/174 (20.1) | 30/93 (32.3) | 65/267 (24.3) | 22/174 (12.6) | 24/93 (25.8) | 46/267 (17.2) | 37/174 (21.3) | 11/93 (11.8) | 48/267 (18.0) |
| 2023-Dec | 43/129 (33.3) | 29/92 (31.5) | 72/221 (32.6) | 43/129 (33.3) | 18/92 (19.6) | 61/221 (27.6) | 27/129 (20.9) | 34/92 (37.0) | 61/221 (27.6) | 20/129 (15.5) | 32/92 (34.8) | 52/221 (23.5) | 21/129 (16.3) | 10/92 (10.9) | 31/221 (14.0) |
| 2024-Jan | 47/153 (30.7) | 32/88 (36.4) | 79/241 (32.8) | 52/153 (34.0) | 18/88 (20.5) | 70/241 (29.0) | 35/153 (22.9) | 29/88 (33.0) | 64/241 (26.6) | 30/153 (19.6) | 29/88 (33.0) | 59/241 (24.5) | 21/153 (13.7) | 13/88 (14.8) | 34/241 (14.1) |
| 2024-Feb | 73/136 (53.7) | 31/62 (50.0) | 104/198 (52.5) | 64/136 (47.1) | 18/62 (29.0) | 82/198 (41.4) | 28/136 (20.6) | 18/62 (29.0) | 46/198 (23.2) | 20/136 (14.7) | 14/62 (22.6) | 34/198 (17.2) | 33/136 (24.3) | 12/62 (19.4) | 45/198 (22.7) |
| 2024-Mar | 83/178 (46.6) | 41/119 (34.5) | 124/297 (41.8) | 74/178 (41.6) | 23/119 (19.3) | 97/297 (32.7) | 41/178 (23.0) | 44/119 (37.0) | 85/297 (28.6) | 26/178 (14.6) | 37/119 (31.1) | 63/297 (21.2) | 44/178 (24.7) | 16/119 (13.4) | 60/297 (20.2) |
| 2024-Apri | 90/215 (41.9) | 59/131 (45.0) | 149/346 (43.1) | 81/215 (37.7) | 34/131 (26.0) | 115/346 (33.2) | 45/215 (20.9) | 35/131 (26.7) | 80/346 (23.1) | 28/215 (13.0) | 30/131 (22.9) | 58/346 (16.8) | 55/215 (25.6) | 26/131 (19.8) | 81/346 (23.4) |
| 2024-May | 85/204 (41.7) | 45/126 (35.7) | 130/330 (39.4) | 74/204 (36.3) | 19/126 (15.1) | 93/330 (28.2) | 45/204 (22.1) | 34/126 (27.0) | 79/330 (23.9) | 30/204 (14.7) | 30/126 (23.8) | 60/330 (18.2) | 38/204 (18.6) | 14/126 (11.1) | 52/330 (15.8) |
| 2024-June | 67/180 (37.2) | 43/135 (31.9) | 110/315 (34.9) | 66/180 (36.7) | 19/135 (14.1) | 85/315 (27.0) | 35/180 (19.4) | 35/135 (25.9) | 70/315 (22.2) | 21/180 (11.7) | 33/135 (24.4) | 54/315 (17.1) | 36/180 (20.0) | 15/135 (11.1) | 51/315 (16.2) |
| 2024-July | 70/196 (35.7) | 47/140 (33.6) | 117/336 (34.8) | 55/196 (28.1) | 29/140 (20.7) | 84/336 (25.0) | 50/196 (25.5) | 35/140 (25.0) | 85/336 (25.3) | 37/196 (18.9) | 29/140 (20.7) | 66/336 (19.6) | 31/196 (15.8) | 13/140 (9.3) | 44/336 (13.1) |
| 2024-Aug | 55/161 (34.2) | 66/159 (41.5) | 121/320 (37.8) | 54/161 (33.5) | 34/159 (21.4) | 88/320 (27.5) | 34/161 (21.1) | 35/159 (22.0) | 69/320 (21.6) | 25/161 (15.5) | 28/159 (17.6) | 53/320 (16.6) | 30/161 (18.6) | 19/159 (11.9) | 49/320 (15.3) |
| 2024-Sep | 68/170 (40) | 94/202 (46.5) | 162/372 (43.5) | 60/170 (35.3) | 67/202 (33.2) | 127/372 (34.1) | 35/170 (20.6) | 37/202 (18.3) | 72/372 (19.4) | 24/170 (14.1) | 20/202 (9.9) | 44/372 (11.8) | 41/170 (24.1) | 44/202 (21.8) | 85/372 (22.8) |
| 2024-Oct | 68/183 (37.2) | 97/201 (48.3) | 165/384 (43.0) | 64/183 (35.0) | 60/201 (29.9) | 124/384 (32.3) | 28/183 (15.3) | 44/201 (21.9) | 72/384 (18.8) | 12/183 (6.6) | 32/201 (15.9) | 44/384 (11.5) | 34/183 (18.6) | 31/201 (15.4) | 65/384 (16.9) |
| 2024-Nov | 49/123 (39.8) | 63/148 (42.6) | 112/271 (41.3) | 42/123 (34.1) | 33/148 (22.3) | 75/271 (27.7) | 22/123 (17.9) | 29/148 (19.6) | 51/271 (18.8) | 15/123 (12.2) | 29/148 (19.6) | 44/271 (16.2) | 20/123 (16.3) | 26/148 (17.6) | 46/271 (17.0) |
| 2024-Dec | 43/120 (35.8) | 60/141 (42.6) | 103/261 (39.5) | 34/120 (28.3) | 35/141 (24.8) | 69/261 (26.4) | 18/120 (15.0) | 33/141 (23.4) | 51/261 (19.5) | 12/120 (10.0) | 26/141 (18.4) | 38/261 (14.6) | 21/120 (17.5) | 23/141 (16.3) | 44/261 (16.9) |
| 2025-Jan | 46/114 (40.4) | 39/114 (34.2) | 85/228 (37.3) | 48/114 (42.1) | 21/114 (18.4) | 69/228 (30.3) | 27/114 (23.7) | 34/114 (29.8) | 61/228 (26.8) | 14/114 (12.3) | 31/114 (27.2) | 45/228 (19.7) | 20/114 (17.5) | 14/114 (12.3) | 34/228 (14.9) |
| 2025-Feb | 41/99 (41.4) | 46/105 (43.8) | 87/204 (42.6) | 37/99 (37.4) | 38/105 (36.2) | 75/204 (36.8) | 28/99 (28.3) | 39/105 (37.1) | 67/204 (32.8) | 19/99 (19.2) | 28/105 (26.7) | 47/204 (23.0) | 15/99 (15.2) | 14/105 (13.3) | 29/204 (14.2) |
| 2025-Mar | 45/120 (37.5) | 75/152 (49.3) | 120/272 (44.1) | 40/120 (33.3) | 47/152 (30.9) | 87/272 (32.0) | 24/120 (20.0) | 39/152 (25.7) | 63/272 (23.2) | 14/120 (11.7) | 28/152 (18.4) | 42/272 (15.4) | 15/120 (12.5) | 21/152 (13.8) | 36/272 (13.2) |
| 2025-Apr | 75/156 (48.1) | 73/172 (42.4) | 148/328 (45.1) | 57/156 (36.5) | 59/172 (34.3) | 116/328 (35.4) | 23/156 (14.7) | 38/172 (22.1) | 61/328 (18.6) | 9/156 (5.8) | 29/172 (16.9) | 38/328 (11.6) | 20/156 (12.8) | 27/172 (15.7) | 47/328 (14.3) |

Note: Data are reported as n (%).

Abbreviations: EA, early antigen; EBNA, Epstein–Barr nuclear antigen; EBV, Epstein–Barr virus; VCA, viral capsid antigen

**Table S2:** Age-specific distribution and positivity rates of serum EBV antibodies and plasma EBV DNA.

| **Age (year)** | **EBV-DNA (%)** | | | **VCA-IgM (%)** | | | **VCA-IgG (%)** | | | **EBN-IgG (%)** | | | **EA-IgG (%)** | | |
| --- | --- | --- | --- | --- | --- | --- | --- | --- | --- | --- | --- | --- | --- | --- | --- |
|  | **Male** | **Female** | **Total** | **Male** | **Female** | **Total** | **Male** | **Female** | **Total** | **Male** | **Female** | **Total** | **Male** | **Female** | **Total** |
| **<0.5** | 0/52  (0) | 0/7  (0) | 0/59  (0) | 0/38  (0) | 0/6  (0) | 0/44  (0) | 20/38 (52.6) | 1/6  (16.7) | 21/44 (47.7) | 29/38 (76.3) | 2/6 (33.3) | 31/44 (70.5) | 3/38 (7.9) | 1/6 (16.7) | 4/44  (9.1) |
| **0.5 to <1** | 8/116 (6.9) | 16/146 (11.0) | 24/262 (9.2) | 4/88 (4.5) | 5/137 (3.6) | 9/225  (4.0) | 4/88  (4.5) | 7/137 (5.1) | 11/225 (4.9) | 5/88 (5.7) | 5/137 (3.6) | 10/225 (4.4) | 4/88 (4.5) | 9/137 (6.6) | 13/225 (5.8) |
| **1 to <3** | 221/660 (33.5) | 420/1238 (33.9) | 641/1898 (33.8) | 144/494 (29.1) | 418/1050 (39.8) | 562/1544 (36.4) | 84/494 (17.0) | 148/1050 (14.1) | 232/1544 (15.0) | 61/494 (12.3) | 93/1050 (8.9) | 154/1544 (10.0) | 72/494 (14.6) | 140/1050 (13.3) | 212/1544 (13.7) |
| **3 to <6** | 660/1251 (52.8) | 1047/2215 (47.3) | 1707/3466 (49.2) | 462/895 (51.6) | 1084/1708 (63.5) | 1546/2603 (59.4) | 292/895 (32.6) | 413/1708 (24.2) | 705/2603 (27.1) | 193/895 (21.6) | 182/1708 (10.7) | 375/2603 (14.4) | 189/895 (21.1) | 374/1708 (21.9) | 563/2603 (21.6) |
| **6 to <9** | 419/830 (50.5) | 552/1226 (45.0) | 971/2056 (47.2) | 279/600 (46.5) | 538/880 (61.1) | 817/1480 (55.2) | 256/600 (42.7) | 314/880 (35.7) | 570/1480 (38.5) | 195/600 (32.5) | 186/880 (21.1) | 381/1480 (25.7) | 152/600 (25.3) | 244/880 (27.7) | 396/1480 (26.8) |
| **≥9** | 167/754 (22.1) | 186/656 (28.4) | 353/1410 (25.0) | 99/549 (18.0) | 192/489 (39.3) | 291/1038 (28.0) | 373/549 (67.9) | 274/489 (56.0) | 647/1038 (62.3) | 367/549 (66.8) | 238/489 (48.7) | 605/1038 (58.3) | 75/549 (13.7) | 105/489 (21.5) | 180/1038 (17.3) |
| **Total** | 1475/3663 (40.3) | 2221/5488 (40.5) | 3696/9151 (40.4) | 988/2664 (37.1) | 2237/4270 (52.4) | 3225/6934 (46.5) | 1029/2664 (38.6) | 1157/4270 (27.1) | 2186/6934 (31.5) | 850/2664 (31.9) | 706/4270 (16.5) | 1556/6934 (22.4) | 495/2664 (18.6) | 873/4270 (20.4) | 1368/6934 (19.7) |

Note: Data are reported as n (%).

Abbreviations: EA, early antigen; EBNA, Epstein–Barr nuclear antigen; EBV, Epstein–Barr virus; VCA, viral capsid antigen

**Table S3:** Positive rates of patients with EBV primary infection, past infection and reactivation by age group.

| **Age(year)** | **Primary infection (n= 3156)** | | |  | **Past infection (n=1290)** | | |  | **Reactivated infection (n=323)** | | |
| --- | --- | --- | --- | --- | --- | --- | --- | --- | --- | --- | --- |
|  | **Male** | **Female** | **Total** |  | **Male** | **Female** | **Total** |  | **Male** | **Female** | **Total** |
| **<0.5** | 1/38 (2.6) | 0/6 (0) | 1/44 (2.3) |  | 30/38 (78.9) | 1/6 (16.7) | 31/44 (70.5) |  | 2/38 (5.3) | 1/6 (16.7) | 3/44 (6.8) |
| **0.5 to <1** | 6/88 (6.8) | 9/137 (6.6) | 15/225 (6.7) |  | 4/88 (4.5) | 4/137 (2.9) | 8/225 (3.6) |  | 1/88 (1.1) | 3/137 (2.2) | 4/225 (1.8) |
| **1 to <3** | 146/494 (29.6) | 414/1050 (39.4) | 560/1544 (36.3) |  | 50/494 (10.1) | 67/1050 (6.4) | 117/1544 (7.6) |  | 18/494 (3.6) | 35/1050 (3.3) | 53/1544 (3.4) |
| **3 to <6** | 447/895 (49.9) | 1073/1708 (62.8) | 1520/2603 (58.4) |  | 154/895 (17.2) | 135/1708 (7.9) | 289/2603 (11.1) |  | 48/895 (5.4) | 56/1708 (3.3) | 104/2603 (4) |
| **6 to <9** | 271/600 (45.2) | 525/880 (59.7) | 796/1480 (53.8) |  | 163/600 (27.2) | 148/880 (16.8) | 311/1480 (21) |  | 36/600 (6) | 42/880 (4.8) | 78/1480 (5.3) |
| **≥9** | 89/549 (16.2) | 175/489 (35.8) | 264/1038 (25.4) |  | 334/549 (60.8) | 200/489 (40.9) | 534/1038 (51.4) |  | 39/549 (7.1) | 42/489 (8.6) | 81/1038 (7.8) |

Note: Data are reported as n (%).
